# Intra-Articular versus Subacromial Corticosteroid Injection for the Treatment of Adhesive Capsulitis: A Meta-Analysis and Systematic Review

**DOI:** 10.1155/2019/1274790

**Published:** 2019-10-15

**Authors:** Xiaoke Shang, Zhong Zhang, Xuelin Pan, Jian Li, Qi Li

**Affiliations:** ^1^Department of Orthopedic Surgery, People's Hospital of Ningxia Hui Autonomous Region, Yinchuan 750000, China; ^2^Department of Orthopedic Surgery, West China Hospital of Sichuan University, Sichuan University, Chengdu, Sichuan 610041, China

## Abstract

**Background:**

Adhesive capsulitis is one of the most well-known causes of pain and stiffness of the shoulder. Corticosteroid injections have been used for many years. However, it is still controversial where corticosteroid should be injected, whether subacromial or intra-articular.

**Objective:**

The objective of this meta-analysis was to compare the effects of intra-articular (IA) and subacromial (SA) corticosteroid injections for the treatment of adhesive capsulitis.

**Materials and Methods:**

Four foreign databases and two Chinese databases were searched for RCTs and quasi-RCTs involving the comparison of IA and SA corticosteroid injection for the treatment of adhesive capsulitis. The Cochrane risk of bias tool and PEDro score were used to evaluate the quality of the studies. The primary clinical outcomes including VAS, Constant score, ASES score, and ROM were collected. The secondary outcome of corticosteroid-related adverse reactions was also compared between the two groups. The results were evaluated and compared at five time points. Subgroup analyses were performed to further explore the differences between groups.

**Results:**

Eight RCTs and one quasi-RCT, involving 512 participants, were identified and included in this meta-analysis. All studies were of low risk of bias and medium-high quality with the PEDro score ≥5 points. The pooled effect showed that there was no significant difference in the primary outcomes between IA injection and SA injection, with an exception of VAS at 2-3 weeks (*P*=0.02) and ROM of internal rotation at 8–12 weeks (*P*=0.02). According to the results of subgroup analyses, the differences of VAS and ROM of internal rotation did not last beyond the 2-3-week time period. Additionally, SA injection had the advantage of avoiding adverse reactions from the corticosteroid, especially in avoiding a large fluctuation of serum blood glucose levels.

**Conclusions:**

When corticosteroid injection is used to treat adhesive capsulitis, both injection sites can be selected. However, due to the scarcity of related studies, more rigorous trials are needed to confirm the current findings.

## 1. Introduction

Adhesive capsulitis of the shoulder, clinically known as “frozen shoulder,” is one of the most well-known causes of pain and stiffness of the shoulder; it usually affects people over the age of 50 years old and is therefore also known as “50 shoulder” in China [1]. Due to the complex pathophysiological process, in 1934, Codman said, “This is a class of cases which I find it difficult to define.” [2]. Except for secondary adhesive capsulitis, the aetiology of idiopathic adhesive capsulitis has not been fully elucidated. Published studies have revealed that adhesive capsulitis is a diffuse inflammatory process, affecting nearly all the periarticular soft tissue components, such as the joint capsule, subacromial bursae, coracohumeral ligament, rotator interval, axillary recess, and the biceps tendon sheath [3, 4]. Due to its strong anti-inflammatory effect, local corticosteroid injection is universally used for the treatment of adhesive capsulitis and has proven to be beneficial for pain relief and function improvement. The injection site is an important factor affecting the effect of hormone therapy. Misinjecting beyond the intended site will not only fail to achieve the desired therapeutic effect but also weaken the tendon strength if injected into the rotator cuff tendon. Repeated puncture could also lead to iatrogenic cartilage injury.

The injection sites of corticosteroid in the treatment of adhesive capsulitis mainly include intra-articular (IA) injection and subacromial (SA) injection. IA injection is the injection type chosen by many doctors, but it has been reported that the accuracy rate ranges only from 26.8% to 40%, without the assistant of image guidance [5, 6]. Regardless of how the injection technique is modified, injection beyond the target area and even outside the joint capsule can still be seen frequently [7]. By contrast, SA injection requires less precision in technique due to the superficial location of the subacromial space. Lho's et al. study [8] found that in addition to the effect on the joint capsules, there was also an overexpression of inflammatory factors in the subacromial bursae of the frozen shoulder. SA injection has been used more frequently in cases of subacromial bursitis, rotator cuff disorders, impingement syndrome, and diagnostic testing rather than in adhesive capsulitis. When IA injection is unresponsive for adhesive capsulitis, some doctors then consider SA injection [9]. Therefore, the optimal site for corticosteroid injection in the treatment of adhesive capsulitis has not been well established. To our knowledge, although there have been several systematic reviews and meta-analyses published in recent years on corticosteroid injection for adhesive capsulitis; none of these meta-analyses specifically compared IA injection with SA injection [10–12]. The purpose of this meta-analysis was not only to compare the clinical effects of IA corticosteroid injection and SA corticosteroid injection in the treatment of adhesive capsulitis more comprehensively, but also to obtain more specific and reliable results through subgroup analyses. It is hypothesized that no significant difference will be found between IA injection and SA injection in pain relief or recovery of function of the shoulder joint, but SA injection may have some advantages over IA injection.

## 2. Materials and Methods

### 2.1. Search Strategy

This meta-analysis was conducted in and is formatted based upon the guidelines of PRISMA (Preferred Reporting Items for Systematic Reviews and Meta-Analyses), which aims to provide a protocol to produce more standardized and comprehensive systematic reviews. Moreover, this study has also been reported in line with AMSTAR (Assessing the methodological quality of systematic reviews) Guidelines. Two authors systematically searched four foreign databases (PubMed, EMBASE, Cochrane Database, and Web of Science) and two Chinese databases (Wan Fang and China National Knowledge Internet) from the project inception to March 30, 2019 with the assistance of a trained librarian, who was skilled in searching medical literature.

The search strategy used various combinations of the search terms (“frozen shoulder,” “stiff shoulder,” “Dupuytren disease,” “periarthritis,” “steroid,” “glucocorticoid”) and the key words (“adhesive capsulitis,” “corticosteroid,” “Intra-articular,” “subacromial,” “injection”). When searching Chinese databases, search terms also included “50 shoulder” and “fifty shoulder.” In addition, reference lists of included articles were manually reviewed to identify any additional relevant articles not captured during the original search.

### 2.2. Identification of Eligibility

The initial screening and study selection were limited to randomized controlled trials (RCTs) and quasi-RCTs with level I-II evidence that specifically compared the outcomes between IA injection and SA corticosteroid injection for the treatment of adhesive capsulitis. In addition to the subgroups of IA injection and SA injection for the treatment of adhesive capsulitis, if a study involved other subgroups such as RI (Rotator Interval) injection or physical therapy, these studies were also included. In such cases, we only extracted data related to the subgroups of IA injection and SA injection. The included literature was further limited to human trials and English-language-only or Chinese-language-only publications. No restrictions were placed on the publication date.

### 2.3. Study Selection

Study selection was conducted using the predefined inclusion criteria and exclusion criteria. To qualify for selection, the studies had to meet the following inclusion criteria: (1) RCTs or quasi-RCTs comparing IA injection and SA injection of corticosteroid for the treatment of adhesive capsulitis; (2) published in peer-reviewed journals; (3) included more than one kind of outcome assessment parameters, such as visual analogue scale (VAS) for pain, Constant score, range of motion (ROM), Shoulder Pain and Disability Index (SPADI), and American Shoulder and Elbow Surgeon (ASES) score; (4) full text and the data were available.

Studies were excluded if they were as follows: (1) review articles, basic science studies including cadaver studies, comments including editorial articles, protocols or letters; (2) regarding shoulder pain due to other causes rather than idiopathic adhesive capsulitis, such as rotator cuff tears, calcific tendonitis, hemiplegia, or cervical radiculopathy; (3)studies comparing the effect of corticosteroid injection with other medication (nonsteroidal anti-inflammatory drugs, NSAIDs), acupuncture, physiotherapy, arthroscopic release, or hydrodilatation; (4) studies related to the comparison of different types of corticosteroid and different doses of corticosteroid, rather than different approaches of administration; (5) studies with a follow-up of less than 2 weeks; (6) abstract publications only.

After removing the duplicate studies, two authors independently reviewed the titles and abstracts, followed by the full text as necessary and excluded those that were obviously unqualified studies. Any disagreement was discussed under the guidance of a senior reviewer until a consensus was reached.

### 2.4. Data Extraction and Outcome Measures

Data from the included studies were extracted independently by two reviewers and verified by a senior reviewer. Using a predefined data collection form which had been pilot tested before use in this meta-analysis, data items recorded from each eligible study included the following: general information about the study (authors, publication year, journal, country), study design and the level of evidence, the inclusion criteria and exclusion criteria, the baseline characteristics of the participants (age, gender, mean duration of symptoms, diabetes status, shoulder dominance), interventions (groups of the study, approaches and sites of the drug administered, frequency of injection, and image-guided or landmark-guided injection), cointerventions (medication including NSAIDs and other analgesics, home exercise, physical therapy), and all of the clinical outcomes.

The primary outcome measurements included pain relief using VAS, function improvement using Constant score and ASES score, and shoulder activity using ROM in various directions. The secondary outcomes included average time for pain relief, number of patients who were lost to follow-up and withdrawn from the study, complications such as glucose fluctuation, and adverse events of the corticosteroid.

Further data processing required data consistency, so it was often necessary to combine the data or convert the data to the form of mean and standard deviation based on Cochrane Handbook (available online at http://www.cochrane.org). If the data were not available in the original article, we extrapolated the data from the supplemental illustrations or contacted the authors by email to request them.

### 2.5. Methodological Quality Assessment

The risk of bias of the included studies was conducted independently by two reviewers according to the Cochrane Collaboration's guidelines [13] for RCTs. These guidelines cover seven items such as random sequence generation, allocation concealment, blinding of participants, blinding of personnel and outcome assessment, incomplete outcome data, selective reporting, and other biases. Each item can be further classified as “low risk,” “unclear risk,” and “high risk” of bias.

As a supplement, we also used an 11-Item Physiotherapy Evidence Database (PEDro) scale described by Maher et al. [14] to assess the methodological quality of the included studies because it can quantify the quality of the literature, specifically for RCTs and quasi-RCTs. Only median to high-quality (PEDro score ≥5) studies were considered for inclusion.

### 2.6. Statistical Analysis

Statistical analyses were completed using the Review Manager (Version: RevMan 5.3). For continuous data, standardized mean differences (SMD) with associated 95% confidence intervals (CI) were calculated to determine the pooled effect of pain relief and function improvement after corticosteroid injection between the two locations for drug delivery. The outcomes used for analyses were not dichotomous data. Heterogeneity was determined using a chi-squared test and *I*^2^ statistic. *I*^2^ values of 25%, 50%, and 75% were deemed as low, medium, and high heterogeneity, respectively. Statistical heterogeneity was established as a *P* value <0.05 or an *I*^2^ value >50%. If there was statistical heterogeneity among the included studies, a random-effects model (REM) was used; otherwise, a fixed-effects model (REM) was selected. When statistical heterogeneity was present, several exploratory sensitivity analyses were conducted by excluding the studies one by one, then judging the stability of the results. Subgroup analyses were performed when necessary in order to obtain a solid conclusion.

## 3. Results

### 3.1. Search Results

A total of 2236 studies were identified from the initial electronic search, of which 197 studies were obtained from the Chinese databases. After removing the duplicated studies and unrelated studies, 394 studies qualified for the further analysis. Through screening the titles and abstracts, 339 studies were excluded since they were irrelevant to the subject. Upon further scrutiny of the 55 remaining studies, 46 studies were excluded according to the inclusion criteria and exclusion criteria: the control groups of 26 studies did not meet the inclusion criteria, 17 studies were basic science studies of adhesive capsulitis, 1 study had incomplete data, and 2 studies were letters with comments.

**Figure 1:**
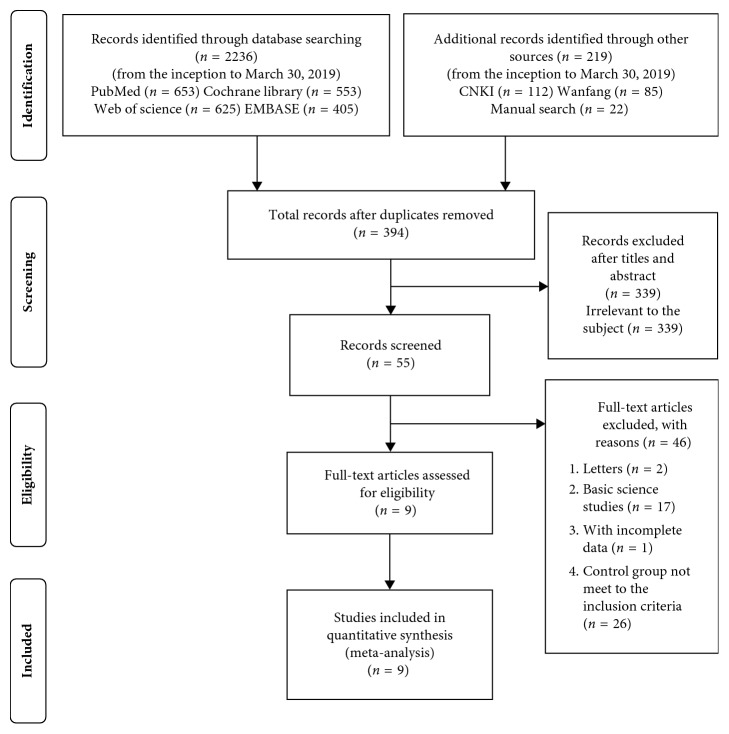
Flow chart of study selection according to the PRISMA Guidelines.

Ultimately, we included 8 RCTs [2, 15–21] and 1 quasi-RCT [22] in this meta-analysis. All of these studies included level I-II evidence, except for one study [22] that had an unclear level. Details of the literature screening process are depicted in Figure 1.

### 3.2. Studies Characteristics

The general characteristics of the 9 included studies are presented in Table 1. All included studies met the inclusion criteria and exclusion criteria except the study by Khallaf et al. [22], which only met the exclusion criteria. Within these 9 eligible studies, a total of 512 patients were used in the comparison between IA injection and SA injection of corticosteroid injection for the treatment of adhesive capsulitis; six [2, 15, 17, 19, 21, 22] out of these 9 studies included additional groups for comparison such as rotator interval (RI) injection and hydrodilatation groups. Five studies [2, 16, 17, 19, 21] expounded the process of patients' screening. In addition, 2 studies [19, 21] restricted the stage of adhesive capsulitis to freezing stage, 2 studies [18, 22] were restricted to freezing stage and frozen stage, 1 study [2] was restricted to frozen stage and thawing stage, and 4 studies [2, 15, 16, 20] did not clearly explain the stages of adhesive capsulitis among their participants. All but one [18] of the included studies reported the baseline characteristics of their participants, which covered the following items with no significant differences among groups: age, gender, mean duration of symptoms, shoulder dominance, and systemic diseases (such as diabetes status, thyroid disease and heart disease).

All patients in the included studies were followed up before corticosteroid injection, and the longest follow-up time was 24 weeks after injection. We further divided the follow-up period into 5 phases (preinjection, 2 to 3 weeks, 4 to 6 weeks, 8 to 12 weeks, and 16 to 24 weeks) with the purpose of assessing the effect of corticosteroid injection between these two approaches in more detail.

### 3.3. Drugs and Technology for Corticosteroid Injection

All included studies reported the composition and dosage of the corticosteroid mixture. The corticosteroid selected in 5 studies [2, 16, 17, 19, 21] was 1 mL of 40 mg/mL triamcinolone, and the other four studies [15, 18, 20, 22] used 1 ml of 40 mg/mL methylprednisolone acetate. Except for one study by Goyal et al. [20], lidocaine was included in the corticosteroid mixture of the included studies, with a concentration of 1-2% and a range of dose of 2–4 ml. Normal saline with a volume of 4-5 ml was mentioned in the corticosteroid mixture of 2 studies [16, 19]. In summary, the maximum volume of the corticosteroid mixture was 10 ml in Yoon's et al. study [19], and the minimum volume was 1 ml in Soha's et al. study [22].

Regarding the IA injection, 3 studies [15, 18, 19] selected the anterior approach while the other 6 studies [2, 16, 17, 20–22] chose the posterior approach. For the SA injection, 6 studies [15, 16, 18, 20–22] selected the lateral approach, 2 studies [17, 19] selected the posterior approach and 1 study [2] selected the superior approach. Regardless of the use of IA or SA injection, 7 studies [2, 16, 18–22] specified the key technical points and the directions of the needles, whereas the other 2 studies [15, 17] did not report these descriptions in detail. All of the studies adopted an ultrasonography-guided technique during injection except Goyal's study, which used a landmark-guided method and Rizk's study whose procedures were unclear.

### 3.4. Assessment of the Methodological Quality and Risk of Bias

**Figure 2:**
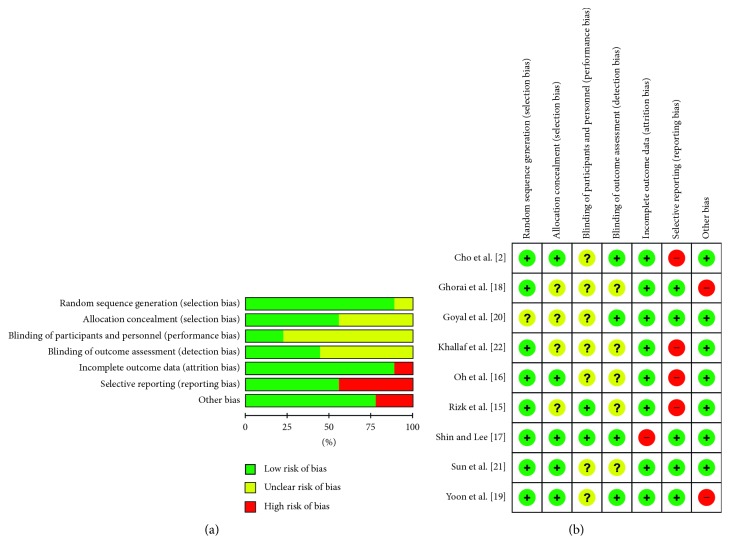
Risk of bias graph (a) and risk of bias summary (b) for each included study.

The risk of bias assessed by the Cochrane tool in each of the included studies is shown in Figure 2. The nine eligible studies included 8 RCTs and 1 quasi-RCT. Seven included studies [2, 16–19, 21, 22] were used to conduct the power analysis and sample size calculations when designing the trials, with the aim of improving the sensitivity to detect differences between groups.

Four studies [2, 16, 17, 19] adopted computer-generated random sequences: 1 study [21] used a sealed opaque envelope, 3 studies [15, 18, 20] were described as “randomized” but did not include detailed methods, and 1 study [22] was unclear as to its randomization procedures. Although only one study [2] explicitly described blinding both patients and assessors, 5 studies [2, 19] conducted blinding to the outcome assessors. In addition, 4 studies [2, 17, 19, 21] described allocation concealment in detail. All of the studies clearly reported follow-up results to avoid reporting bias, although the follow-up intervals were not consistent. Six studies [2, 15, 16, 18, 19, 21] mentioned the patients who had been lost to follow-up or withdrawn from their studies, which could have prevented attrition bias. However, the shortcoming of these 6 studies was the lack of intention-to-treat (ITT) analysis.

According to the PEDro score, the methodological quality scores varied from 5 to 9 out of a possible score of 10 (Table 2). All of the included studies were considered to be of medium-high quality.

### 3.5. Result of the Meta-Analysis

#### 3.5.1. Primary Outcome Measurements of the VAS, Constant Score, ASEA Score, and ROM

Table 3 provides the detailed information of the meta-analysis on the primary outcome measurements of pain release by the VAS score, Constant score, ASEA score, and the ROM of the shoulder joint (including forward flexion, external rotation, abduction, and internal rotation). Based on the data at baseline for each primary outcome measurement, there was no significant difference in the pooled effect of the meta-analysis between IA injection and SA injection. This meant that the patients were typically represented, and the data could be used for further comparisons.

**Figure 3:**
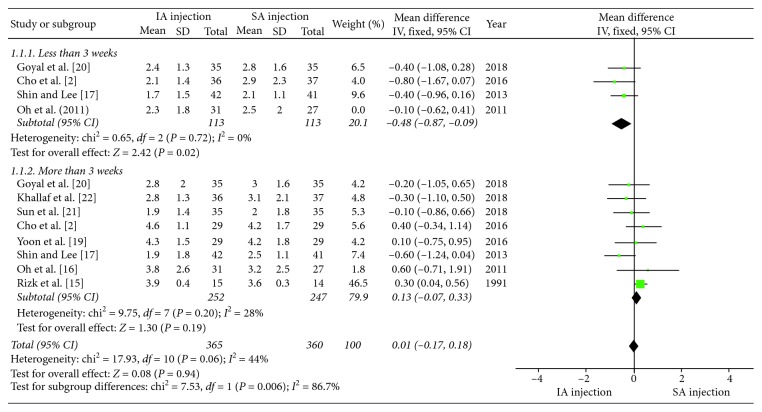
Forest plot of pain for associated subgroup analysis (≤3 weeks and >3 weeks). The total pooled effect was not statistically significant between the IA injection and SA injection groups (*P*=0.06).

Furthermore, no significant differences were seen at other time points for each primary outcome measurement between the two corticosteroid injection approaches, with the exception of pain at 2 to 3 weeks, and ROM of internal rotation at 8 to 12 weeks. Similar to the study by Oh et al., pain relief measured by VAS was greater with IA injection than SA injection at 2 to 3 weeks (*P*=0.02). Subgroup analyses of different categories (≤3 weeks and >3 weeks) for the VAS score showed that the pooled effect of the meta-analysis did not find statistically significant differences between the IA injection and SA injection (MD = 0.01, 95% CI: −0.07–0.33, *P*=0.94). A forest plot of pain with associated subgroup analyses is shown in Figure 3.

**Figure 4:**
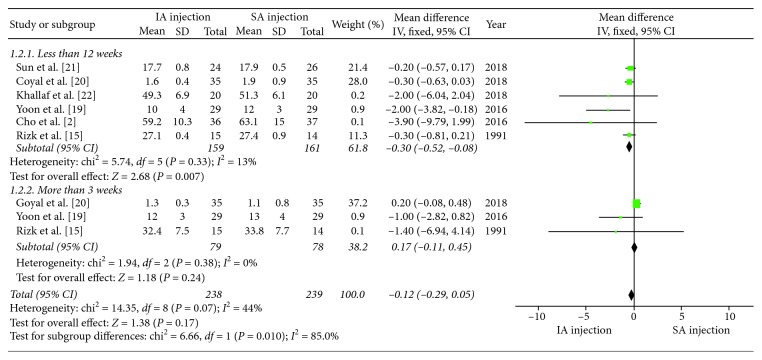
Forest plot for ROM of internal rotation for associated subgroup analysis (≤12 weeks and >12 weeks). The total pooled effect was not statistically significant between the IA injection and SA injection groups (*P*=0.17).

As for the ROM of internal rotation, the SA injection group had greater ROM of internal rotation when compared with the IA injection group at 8 to 12 weeks (*P*=0.02). However, a subgroup analysis yielded a negative result by dividing the follow-up time points into groups of less than 12 weeks and more than 12 weeks. The total pooled effect on the ROM of internal rotation was not significantly different between the IA injection group and SA injection group (MD = −0.12, 95% CI: −0.29–0.05, *P*=0.17). A forest plot for ROM of internal rotation associated subgroup analyses is shown in Figure 4.

**Figure 5:**
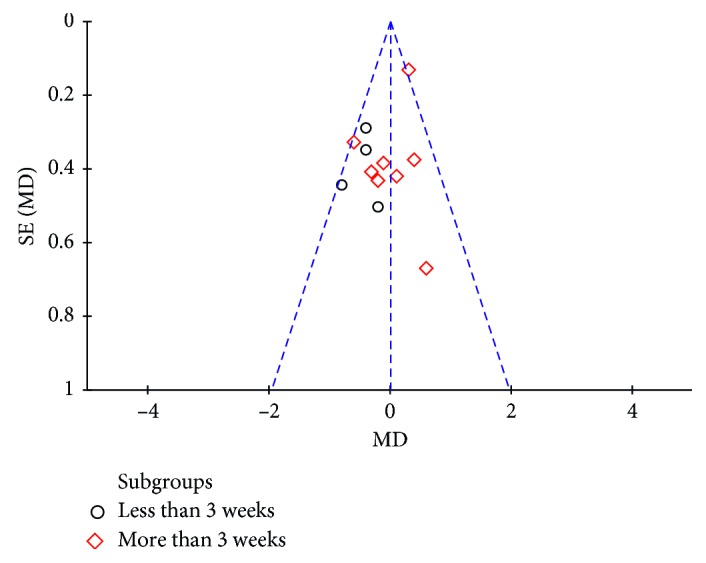
Funnel plots for detecting publication bias related to the subgroup comparisons of pain.

Furthermore, a sensitivity analysis was performed to verify the reliability and stability of the results. After excluding the individual studies one by one, the corresponding pooled results had no obvious fluctuation, indicating that none of the studies seriously affected the final outcome. The funnel plot of the subgroup analysis of pain relief by VAS is shown in Figure 5. The funnel plot's shape is mostly symmetrical, indicating that no significant publication bias was found.

#### 3.5.2. Secondary Outcome Measurements

Only one study by Rizk et al. [15] reported the average time for pain relief; the IA injection group's average time was 2.3 weeks, and the SA injection group's average time was 2.2 weeks. Six studies [2, 15, 16, 18, 19, 21] reported the number of patients who were lost to follow-up and withdrawn from each study, but there was no significant difference in the final pooled effect between the IA injection group and SA injection group (OR = 0.89, 95% CI: 0.47–1.67, *P*=0.71). Five studies [2, 16, 17, 19, 21] showed the adverse events of corticosteroid injection for the treatment of adhesive capsulitis. The total incidence of adverse events related to corticosteroid injection was 4.1% (21 of 512 patients). Three studies [18, 19, 21] further differentiated the numbers of adverse events between the two injection methods. Yoon et al. [19] reported that 2 patients in the IA group and 1 patient in the SA group had mild dizziness and nausea after the injection. Sun et al. [21] reported that 1 patient in each group showed temporary facial flushing within 15 minutes after injection.

With the exception of the study by Ghorai et al. [18], which directly excluded the patients with diabetes, four studies [2, 16, 19, 20] reported the distribution of diabetic patients included in the study, while the other four studies [15, 17, 21, 22] were not clear about the patients' diabetes status. Additionally, in the study by Oh et al. [16], the serum blood glucose levels in the IA group increased from 146+/50 mg/dL before injection to 181+/80 mg/dL at 3 weeks after injection, while in the SA group, the serum blood glucose levels increased from 144+/27 mg/dL to 153+/34 mg/dL. Compared with the IA group, the SA group had smaller fluctuations in the serum blood glucose levels after the corticosteroid injection.

## 4. Discussion

### 4.1. Summary of the Findings

The results of this meta-analysis suggest that there is no significant difference between IA and SA corticosteroid injection for the treatment of adhesive capsulitis based on the analysis of the VAS score, Constant Score, ASEA Score, and ROM in various directions, with the exception of pain relief at 3 weeks and ROM of internal rotation at 12 weeks. These differences were not obvious at other time phases and do not last longer than the specified time phase. Additionally, SA injection has the advantage of avoiding adverse reactions of the corticosteroid. This is of great clinical value, and therefore, SA injection may be more suitable for adhesive capsulitis patients with diabetes mellitus. Our hypothesis was that SA corticosteroid injection has some advantages over IA injection in the treatment of adhesive capsulitis. Our hypothesis has been confirmed to some extent on the basis of this review.

Unlike other reviews [11, 23, 24], we did not divide the follow-up time points into short-term and long-term periods. Rather, we included 5 time points based on the follow-up periods in the included studies. We based this decision on the following considerations. First, there were no clear and generally accepted boundaries between the short-term and the long-term periods in the included studies. Second, the main purpose of hormone therapy for adhesive capsulitis was to eliminate or reduce the inflammatory state of the adhesive capsulitis. Because the exact stage of adhesive capsulitis was not very clear and can overlap with another stage, it was difficult to distinguish which period belonged to the severe inflammatory stage, which period belonged to the mild inflammatory stage, and which stage did not have inflammation. Therefore, we believe that only more detailed analysis will lead to a reliable conclusion.

### 4.2. Comparison with Existing Literature

In the present work, significant differences were found between the IA injection and SA injection in VAS score at 2 to 3 weeks and in ROM of internal rotation at 8 to 12 weeks. Since significant differences were found only at one of the five time points in the VAS score or the ROM of internal rotation, subgroup analyses were conducted at the boundaries of 3 weeks in VAS and at 12 weeks in ROM of internal rotation, but no significant differences were found.

The results of the current meta-analysis are similar to the study by Oh et al. [16], Goyal et al. [20], Cho et al. [2], and Soha et al. [22]. In three [2, 20, 22] of these four studies, SA injection was found to be more effective in improving shoulder mobility, especially the ROM of internal rotation at 12 weeks. The analytical results revealed that the pooled effect was more favourable to the SA injection at less than 12 weeks (*P*=0.007). However, at more than 12 weeks, the pooled effect did not favour either SA injection or IA injection (*P*=0.24). The main explanation for this discrepancy may be that injection into the SA space can more directly reach extra-articular structures such as the rotator interval and coracohumeral ligament, and these structures play an important role in the ROM of internal rotation of the shoulder.

Oh's et al. [16] findings suggested that IA injection may have a better effect on pain relief by VAS at 3 weeks. Some studies [25–27] confirm that adhesive capsulitis is accompanied by the release of inflammatory factors, which are mainly concentrated in the joint cavity, so IA injection of corticosteroid can directly target these inflammatory factors. Consistent with recent meta-analyses [10, 11, 28], IA injection may be more effective in pain relief by VAS in the short term, but the effect was not sustained in the long term.

In a previous systematic review [29], Shah found the adverse reactions associated with corticosteroid injection for the treatment of adhesive capsulitis which included blood glucose fluctuation, irregular menstrual bleeding (10.5%), dizziness, facial flushing (12.5–20%), and rash (4%). However, Shah did not further separate IA injections and SA hormone injections from the total adverse reactions. In the present meta-analysis, we found that the total incidence of adverse reactions was 4.1%. IA injection seemed to involve more adverse events and caused more blood glucose fluctuations than SA injection.

In a recent study, Desai et al. [30] found that subacromial corticosteroid injection increases the risk of revision after rotator cuff repair. This happened mainly with multiple injections, not just a single injection. However, no similar conclusions have been reported in our meta-analysis, and further studies are needed to determine whether subacromial corticosteroid injection can cause an increase in rotator cuff tears. Additionally, of the included studies, 7 studies included corticosteroid injection conducted under the guidance of ultrasonography, and only 2 studies conducted corticosteroid injection under the guidance of landmarks. Therefore, the data related to the injection accuracy comparing these two approaches were not included in this study. We believe that the accuracy of these two injection methods were both very high under the guidance of ultrasonography, and the difference might be very slight.

### 4.3. Limitations of This Meta-Analysis

As in most meta-analyses, several possible limitations are worthy of comment. First, although we asked a professional librarian to help with document retrieval, we might not have identified all possible studies such as conference papers, dissertations, etc. Some studies may be omitted because all possible search terms cannot be included. We believe that adhesive capsulitis may have other names in some regions, just as it is called “50 shoulder” in China.

Second, while every effort was comprehensive, deficiencies in statistical analyses, methodology, or data consolidation processing may not reveal more subtle differences between the two groups. For example, not all studies used the same standard for measuring the ROM of internal rotation, so a large data fluctuation was observed among studies. However, no significant differences were found in the other 4 time points apart from the differences observed at 12 weeks. Therefore, the results of the pooled effect in the subgroup analyses were not significantly affected.

Third, due to lack of data related to some confounding factors, the accuracy of the results may be influenced. Possible confounding factors may include the types and dosage of the corticosteroid, the dosage of mixed lidocaine, and the volume of the mixtures. Existing studies [31, 32] have shown that no significant difference is found between a high dose and a low dose of corticosteroid injection for the treatment of adhesive capsulitis. Conversely, one study [33] has shown that a high dose is more effective than a low dose. Kim et al. [34] examined the effect of lidocaine test injection on corticosteroid injection in the treatment of adhesive capsulitis. Before corticosteroid injection, lidocaine injection into the SA space can better identify the source of pain and guide the next corticosteroid injection site. However, since the half-life of lidocaine is short, there are no data regarding the injection of lidocaine alone for the treatment of adhesive capsulitis. In addition, the maximum volume of the corticosteroid mixture was no more than 10 ml. This volume is far less than the usual volume used for hydrodilatation [19, 35] for the treatment of adhesive capsulitis. Furthermore, of the 9 studies included in this meta-analysis, with the exception of two studies [2], corticosteroid injection was conducted under the guidance of imaging. Due to the lack of literature referring to landmark-guided corticosteroid injection, no further subgroup analyses were carried out. In summary, more studies are needed to provide detailed data to verify the impact of these confounding factors on the results.

### 4.4. Implications for Daily Clinical Practice and Future Studies

The results of this study have significant clinical value for the daily clinical practice of doctors. This meta-analysis suggests that in addition to IA injection, doctors can also choose SA corticosteroid injection for the treatment of adhesive capsulitis. To avoid corticosteroid-related adverse reactions, SA injection seems to be a more suitable option, especially for patients with diabetes. Therefore, future studies should design more rigorous trials to draw more reliable conclusions. In addition, for the abovementioned confounding factors, more targeted research should be designed to support our conclusions.

## 5. Conclusions

The results of this meta-analysis confirm that there is a slight difference between IA and SA corticosteroid injection for adhesive capsulitis at a single time phase, but this difference does not last longer than that time period. In addition, SA injection has the advantage of avoiding adverse reactions, especially avoiding large fluctuations of serum blood glucose levels. This finding suggests that SA injection may be considered in adhesive capsulitis patients with diabetes mellitus whose daily blood glucose level is not well controlled. However, due to the scarcity of related studies, more research is needed to verify this conclusion. In view of the limitations discussed earlier, the results need to be interpreted with caution.

## Figures and Tables

**Table 1:** Characteristics of the included studies and overview of corticosteroid injection related to IA and SA injection for adhesive capsulitis.

Study	Study design	Patients analysed (n)	Corticosteroid mixture	Duration of symptoms	Follow-up
Sun et al. [21]	RCT	Total = 50 IA (24)/SA (26)	A single injection: 1 mL of 40 mg/mL triamcinolone +2 mL 2% lidocaine	IA (15.2 ± 5.0) vs. SA (15.1 ± 4.8) weeks	0 (baseline), 4, 8, and 12 weeks after injection

Goyal et al. [20]	Quasi-RCT	Total = 70 IA (35)/SA (35)	40 mg of methylprednisolone acetate	IA (15.6 ± 4.9) vs. SA (14.2 ± 4.4) weeks	Before injection, 3, 6 and 12 weeks and 6 months

Khallaf et al. [22]	RCT	Total = 40 IA (20)/SA (20)	A single injection: 1 ml 40 mg methylprednisolone acetate +1 mL 2% lidocaine	Unclear	Before injection, 12 weeks after injection

Cho et al. [2]	RCT	Total = 73 IA (36)/SA (37)	A single injection: 1 ml 40 mg triamcinolone acetonide +4 mL 1% lidocaine	IA (21.2 ± 14.6) vs. SA (17.4 ± 14.0) weeks	Before injection, 3, 6 and 12 weeks after injection

Yoon et al. [19]	RCT	Total = 58 IA (29)/SA (29)	A single injection: 1 ml 40 mg triamcinolone acetonide +4 mL 2% lidocaine +5 mL normal saline	IA (9 ± 6) vs. SA (9 ± 5) months	Before treatment and 1 month, 3 months, and 6 months

Ghorai et al. [18]	RCT	Total = 51 IA (25)/SA (26)	A single injection: 1 ml 40 mg depotmethyl prednisolone +1 mL 2% lignocaine	Unclear	Before injection, 3, 6 weeks after injection

Shin and Lee [17]	RCT	Total = 83 IA (42)/SA (41)	A single injection: 1 ml 40 mg triamcinolone acetonide +4 mL 2% lidocaine	IA (7.4 ± 3.4) vs. SA (7.7 ± 3.3) months	Before treatment and at 2, 4, 8, 16, and 24 weeks

Oh et al. [16]	RCT	Total = 58 IA (31)/SA (27)	A single injection: 1 ml 40 mg triamcinolone +4 mL 2% lidocaine +4 mL saline	IA (6.2 ± 3.6) vs. SA (6.9 ± 3.4) months	Preinjection and 3, 6, and 12 weeks after injection

Rizk et al. [15]	RCT	Total = 29 IA (15)/SA (14)	Three injections in the same location at intervals of one week: 1 mL 40 mg/mL methylprednisolone +2 mL of 1% lidocaine	Mean 13.2 (range 8–18) weeks	Weekly for 11 weeks and 15 weeks and six months

**Table 2:** Physiotherapy Evidence Database (PEDro) scale was used for the quality evaluation of the included studies.

Study	Random allocation	Concealed allocation	Baseline comparability	Blind subject	Blind clinician	Blind assessor	Adequate follow-up	Intention-to-treat analysis	Between-group analysis	Point estimates and variability	Total score
Sun et al. [21]	1	1	1	0	0	0	1	0	1	1	6
Goyal et al. [20]	0	0	1	0	0	1	1	0	1	1	5
Khallaf et al. [22]	1	0	1	0	0	0	1	0	1	1	7
Cho et al. [2]	1	1	1	1	0	1	1	0	1	1	8
Yoon et al. [19]	1	1	1	1	0	1	1	0	1	1	8
Ghorai et al. [18]	1	0	1	0	0	0	1	0	1	1	7
Shin and Lee [17]	1	1	1	1	0	1	1	0	1	1	8
Oh et al. [16]	1	0	1	1	0	1	1	0	1	1	7
Rizk et al. [15]	1	0	1	1	0	1	1	0	1	1	9

**Table 3:** Comparison of the primary outcomes involving VAS score, Constant score, ASEA score, and the ROM of various directions.

Time phases	Included studies	Chi	*I* ^2^ (%)	*Z*	*P*
Pain by VAS score					
Baseline	①, ②, ③, ④, ⑤, ⑦, ⑧, ⑨	5.57	0	1.69	0.09
2 to 3 weeks	②, ④, ⑦, ⑧	0.65	0	2.42	0.02
4 to 6 weeks	①, ②, ④, ⑦, ⑧, ⑨	7.02	29	0.00	1.00
8 to 12 weeks	①, ②, ③, ④, ⑤, ⑦, ⑧, ⑨	6.66	0	0.80	0.42
16 to 24 weeks	①, ⑤, ⑦, ⑨	4.64	35	0.08	0.93

Constant score					
Baseline	①, ②, ⑤, ⑧	2.18	0	0.60	0.55
2 to 3 weeks	②, ⑧	0.04	0	1.11	0.27
4 to 6 weeks	①, ②, ⑤, ⑧	0.88	0	1.09	0.27
8 to 12 weeks	①, ②, ⑤, ⑧	5.05	41	0.57	0.57
16 to 24 weeks	②, ⑤	0.04	0	0.74	0.46

ASEA score					
Baseline	④, ⑦	0.00	0	0.26	0.79
2 to 3 weeks	④, ⑦	2.55	61	0.92	0.36
4 to 6 weeks	④, ⑦	3.06	67	1.39	0.16
8 to 12 weeks	④, ⑦	3.19	65	1.34	0.16
16 to 24 weeks	⑦	—	—	—	—

Forward flexion					
Baseline	①, ②, ③, ④, ⑤, ⑥, ⑦, ⑨	5.43	0	0.49	0.62
2 to 3 weeks	①, ④, ⑥, ⑨	3.59	17	1.76	0.08
4 to 6 weeks	①, ②, ④, ⑤, ⑥, ⑦, ⑨	12.71	45	0.02	0.98
8 to 12 weeks	①, ②, ③, ④, ⑤, ⑦, ⑨	8.96	33	0.79	0.43
16 to 24 weeks	①, ⑤, ⑦, ⑨	2.84	0	0.13	0.90

External rotation					
Baseline	①, ②, ③, ④, ⑤, ⑥, ⑦, ⑧, ⑨	7.11	0	0.07	0.94
2 to 3 weeks	②, ④, ⑥, ⑦, ⑧, ⑨	4.82	0	0.82	0.41
4 to 6 weeks	①, ②, ④, ⑤, ⑥, ⑦, ⑧, ⑨	13.97	49	1.67	0.09
8 to 12 weeks	①, ②, ③, ④, ⑤, ⑦, ⑧, ⑨	8.91	21	0.35	0.73
16 to 24 weeks	②, ⑤, ⑦, ⑨	1.37	0	1.83	0.07

Abduction					
Baseline	①, ②, ③, ④, ⑥, ⑧, ⑨	4.99	0	0.63	0.53
2 to 3 weeks	②, ④, ⑥, ⑧, ⑨	5.63	29	1.27	0.20
4 to 6 weeks	①, ②, ④, ⑥, ⑧, ⑨	9.26	46	0.35	0.73
8 to 12 weeks	①, ②, ③, ④, ⑧, ⑨	6.41	22	1.23	0.22
16 to 24 weeks	②, ⑨	0.78	0	0.50	0.62

Internal rotation					
Baseline	①, ②, ③, ④, ⑤, ⑨	9.46	47	0.57	0.57
2 to 3 weeks	②, ④, ⑨	0.16	0	0.32	0.75
4 to 6 weeks	①, ②, ④, ⑤, ⑨	1.63	0	0.14	0.89
8 to 12 weeks	①, ②, ③, ④, ⑤, ⑨	11.06	55	2.38	0.02
16 to 24 weeks	②, ⑤, ⑨	1.94	0	1.18	0.24

①Sun et al. [21], ②Goyal et al. [20], ③Khallaf et al. [22], ④Cho et al. [2], ⑤Yoon et al. [19], ⑥Ghorai et al. [18], ⑦Shin and Lee [17], ⑧Oh et al. [16], and ⑨Rizk et al. [15].

## Data Availability

The data used to support the findings of this study are available from the corresponding author upon request.

## Abbreviations

IA: Intra-articular injection
SA: Subacromial injection
IL: Interleukin
TNF-*α*: Tumour necrosis factor-*α*
RCT: Randomized controlled trial
NRCT: Nonrandomized controlled trials
VAS: Visual analogue scale
ROM: Range of motion
PRISMA: Preferred Reporting Items for Systematic Reviews and Meta-Analyses
PED: Physiotherapy Evidence Database
FXM: Random-effects model
REM: Fixed-effects model.
